# A pilot study showing that repeated exposure to stress produces alterations in subsequent responses to anesthetics in rats

**DOI:** 10.1371/journal.pone.0214093

**Published:** 2019-03-25

**Authors:** Lingzhi Wang, Lindsay Holland, Robert Fong, Suhail Khokhar, Aaron P. Fox, Zheng Xie

**Affiliations:** 1 Department of Anesthesia and Critical Care, The University of Chicago, Chicago, Illinois, United States of America; 2 Department of Anesthesia, the Second Affiliated Hospital, Guangzhou Medical University, Guangzhou, P. R. China; 3 University of Michigan, College of Medicine, Ann Arbor, Michigan, United States of America; 4 University of Illinois at Chicago, College of Medicine, Chicago, Illinois, United States of America; 5 Department of Neurobiology, Pharmacology and Physiology, The University of Chicago, Chicago, Illinois, United States of America; Massachusetts General Hospital, UNITED STATES

## Abstract

The repeated use of a drug frequently leads to alterations in the response to that drug. We undertook this study to determine whether multiple exposures to the general anesthetic produced alterations in subsequent exposures to this anesthetic. For this study, adult male rats were anesthetized with 2.5% isoflurane for one hour. The rats were divided into 4 groups of 8 rats each. Groups 1–3 were transported between their homeroom and the anesthesia testing room and were handled in an identical manner weekly for a period of 12 weeks, but were anesthetized on different schedules. Group 1 was anesthetized weekly for 12 weeks, Group 2 on either a 3 or 4 week schedule and Group 3 was anesthetized a single time, at the end of the 12 week period. To receive anesthesia multiple times, animals were transported from their homeroom to the anesthesia location and handled repeatedly. We took into consideration of the frequency of anesthesia exposure and the stress involved. Rats in groups 2 and 3 were placed in the anesthesia chamber, with O_2_ but with no anesthetic, every week when they were not scheduled to receive anesthesia. In Group 4, rats were not transported or handled in any way and stayed in the home room for a period of 12 weeks. Rats in this group were anesthetized once, at the very end of the study. Recovery of the rat’s righting reflex was used to assess the acceleration of recovery time from general anesthesia. Group 1 rats showed dramatically faster emergence from anesthesia after several rounds of anesthesia. Surprisingly, Groups 2 and 3 rats, treated in an identical manner as Group 1, but which were anesthetized on different schedules, also exhibited more rapid emergence from anesthesia, when compared to Group 4 rats, which were never handled or transported prior to a single anesthesia. These results suggest that the stress of transportation and handling altered responsiveness to anesthesia. Our results show that responsiveness to anesthetic agents can change over time outside of the normal developmental changes taking place in rats as they age.

## Introduction

Exposing animals or humans to a variety of drugs alters responses to subsequent exposures to the same drugs. Opioids provide a particularly well studied example. μ-opioid receptors are G-protein coupled receptors. μ-opioid agonists are among the most effective drugs for pain relief. Protracted or repeated activation of these receptors results in tolerance to these drugs [1–3]. It is not clear whether repeated use of volatile anesthetics, the most commonly used agents in anesthesia, can alter the sensitivity of these drugs in future use. One of the main effects of volatile anesthetics is to induce reversible unconsciousness [4]. Unrecognized change in anesthetic sensitivity can lead to untoward events, such as intraoperative awareness and recall. Our current study was initiated to determine whether repeated exposure to the volatile anesthetic isoflurane altered subsequent responses to the same drug. This is a clinically important question as there are patients who receive multiple rounds of anesthesia. In this study, in order to receive anesthesia multiple times, animals were transported from their homeroom to the anesthesia location and handled repeatedly. Repeated transportation and handling may elicit stress in these animals. In humans, patients usually receive anxiolytics, such as midazolam, to reduce stress before they are transported to the operation room. Nonetheless, anticipation of surgery for days prior to the procedure and the procedure itself will result in stress.

There is a significant literature suggesting that stress affects health and alters neuronal function. Chronic stress can result in anxiety, insomnia, high blood pressure and a weakened immune system [5]. Stress can alter the architecture of the developing brain [6]. Short-term stress, lasting just a few hours, impairs synaptic communication that may lead to altered learning and memory [7]. Repeated chronic stress leads to cognitive impairment by altering glutamate receptor expression [8] and by altering long-term potentiation [9]. Elevated levels of corticosterone, the major stress hormone, produces persistent cognitive and emotional changes [10]. Memory appears to be especially prone to disruption by stress [11]. Stress responses may act as a trigger for mental illness [12]. Furthermore, chronic stress may produce fewer neurons and elevated numbers of glial cells; this imbalance may lead to mental disorders [13]. Not surprisingly, surgery and anesthesia appear able to activate the body’s response to stress [14].

In this study, we designed experiments to determine the effects of the frequency and number of anesthesia exposures and the stress altered responses to isoflurane by measuring the reduction in time to emerge from anesthesia. We hypothesize that repeated exposure to isoflurane or/and stress involved may alter the operation of anesthetic agents.

## Material and methods

### Anesthetizing adult rats

All studies on rats were approved by The University of Chicago Animal Use Committee. This manuscript adheres to the applicable Arrive guidelines. All efforts were made to minimize animal suffering, to reduce the number of animals used, and to utilize alternatives to in vivo techniques, if available.

### Experimental design

**Fig 1:**
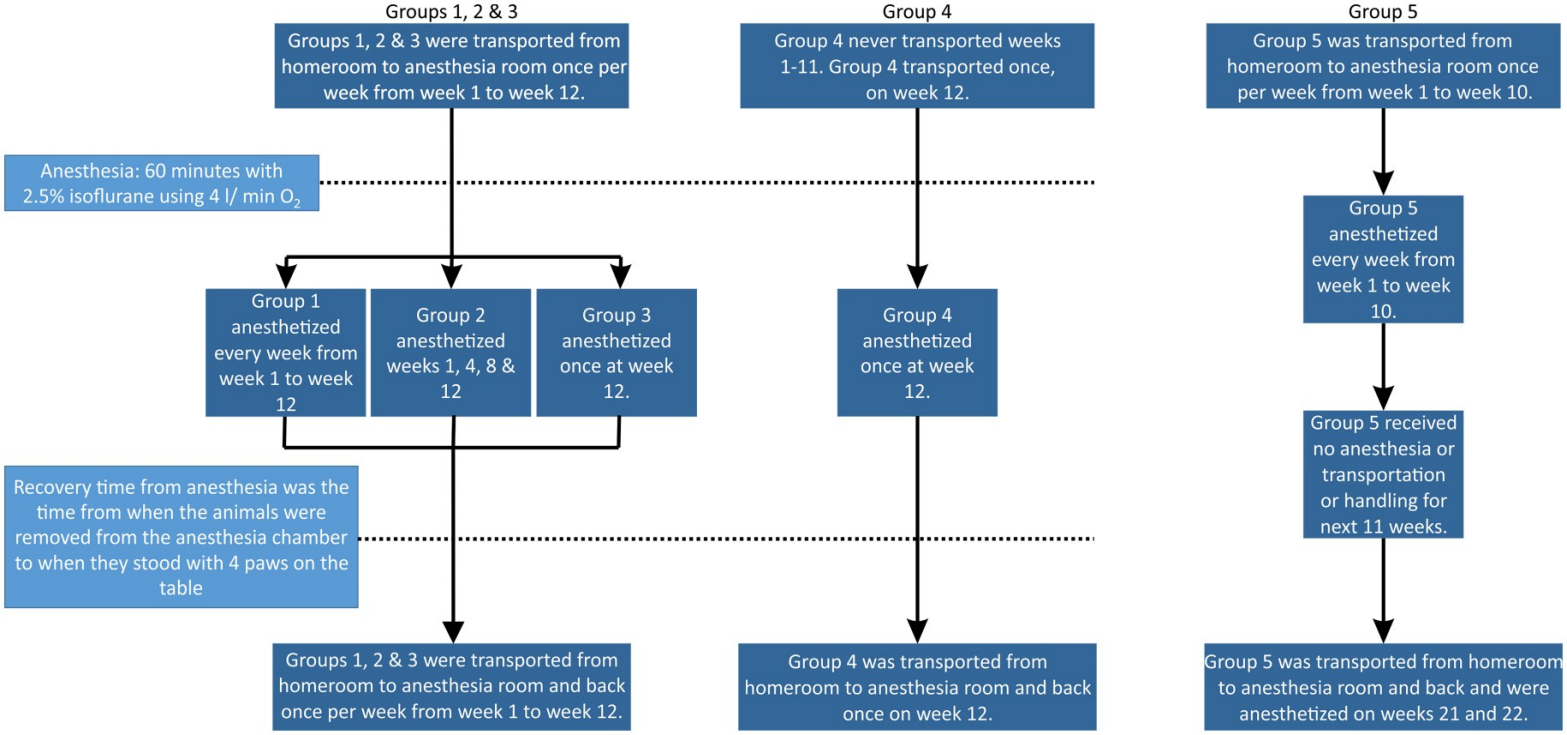
Description of experimental protocol. Flow chart describing handling and anesthesia schedule for the 5 groups of rats tested in Figs 2–5. Each group had 8 rats (n = 8 each).

Adult Sprague Dawley rats (Charles River) arrived in the University of Chicago animal care facility at 10–12 weeks of age weighing 310–400 gm. These rats were housed in the same home room in the animal care facility for 2 weeks before they were used for experiments. They were divided into 4 Groups with 8 rats each in a cohort manner. These rats were taken out from their homeroom on the sub-basement level and transferred to the animal lab on the basement level on the day of experiment. They were returned to their homeroom after the experiment. The description of the handling and anesthesia schedules for Groups 1–4 are shown in Fig 1. In Group 1, the rats were anesthetized once per week (12 total anesthesia sessions). In Group 2, the rats were transported and handled in an identical manner to those in Group 1, but were anesthetized once every 3 or 4 weeks (4 total anesthesia sessions) for a period of 12 weeks. In Group 3, the rats were transported and handled in an identical manner to the rats of Groups 1 and 2, but this group was anesthetized only once at the very end of the 12 week period. Rats in groups 2 and 3 were put into the anesthesia chambers with 4 L O2 for 5 minutes without anesthesia every week even when they were not scheduled to receive anesthesia. This maneuver was similar to the handling the group 1 rats received when they were placed in the anesthesia chamber at the beginning of their scheduled anesthesia. Group 4 (Control) was not transported or handled in any way and stayed in the home room for a period of 12 weeks. Group 4 rats were anesthetized once, at the very end of the study. All rats were housed in the same room in the animal care facility and were cared by the facility staffs except the time when they were transported to the anesthesia room on another floor.

### Isoflurane experiments

Adult rats, weighing 350–700 gm, were placed in a gas-tight anesthesia box (VetEquip, IMPAC6) where they were exposed to 2.5% isoflurane (in 4L/min O_2_) for one hour [15]. During this time, the rats became unconscious and were insensitive to tail pinch. We used high flow (4L/min O2) and one hour isoflurane anesthesia to ensure that isoflurane reached equilibrium in these rats. Anesthesia was then terminated at the end of one hour period and rats were placed on their backs in the middle of a large table. Recovery time from anesthesia was the time from when the animals were removed from the anesthesia chamber to when they stood with 4 paws on the table. The return of the righting reflex in rats is generally considered as a proxy for recovery of consciousness. The anesthesia machine, the anesthesia chambers and the active scavenging system were tested regularly by our certified staff in the animal care center. All experiments were performed in the daytime at the room temperature around 22 °C.

A different group of rats was tested with 3% isoflurane separately (Group 5). These animals were ~2.5 months old at the start of the study described in Fig 1. Otherwise they were handled in the same manner as the rats anesthetized weekly with 2.5% isoflurane. Because the changes observed appear to result from repetitive stress and not repeated exposures to isoflurane, we show experiments using either 2.5% or 3% isoflurane interchangeably.

### Statistical analysis

Data between groups of animals was analyzed with an unpaired T-test. Data within a group was analyzed with a paired T-test. For multiple comparisons, a repeated measure ANOVA was used. Data were expressed in Mean ± SEM. The “*” corresponds to a significant difference in the average emergence values, p <0.05. The “**” denotes p<0.01.

In a previous study [15] we showed that there could be significant variability between groups of rats in their responses to anesthetics, but not within the same group used multiple times. The important findings in this study are within individual groups and the study is appropriately powered for within group comparisons. The sample size may not be sufficient to make definitive statements for between group comparisons.

## Results

**Fig 2:**
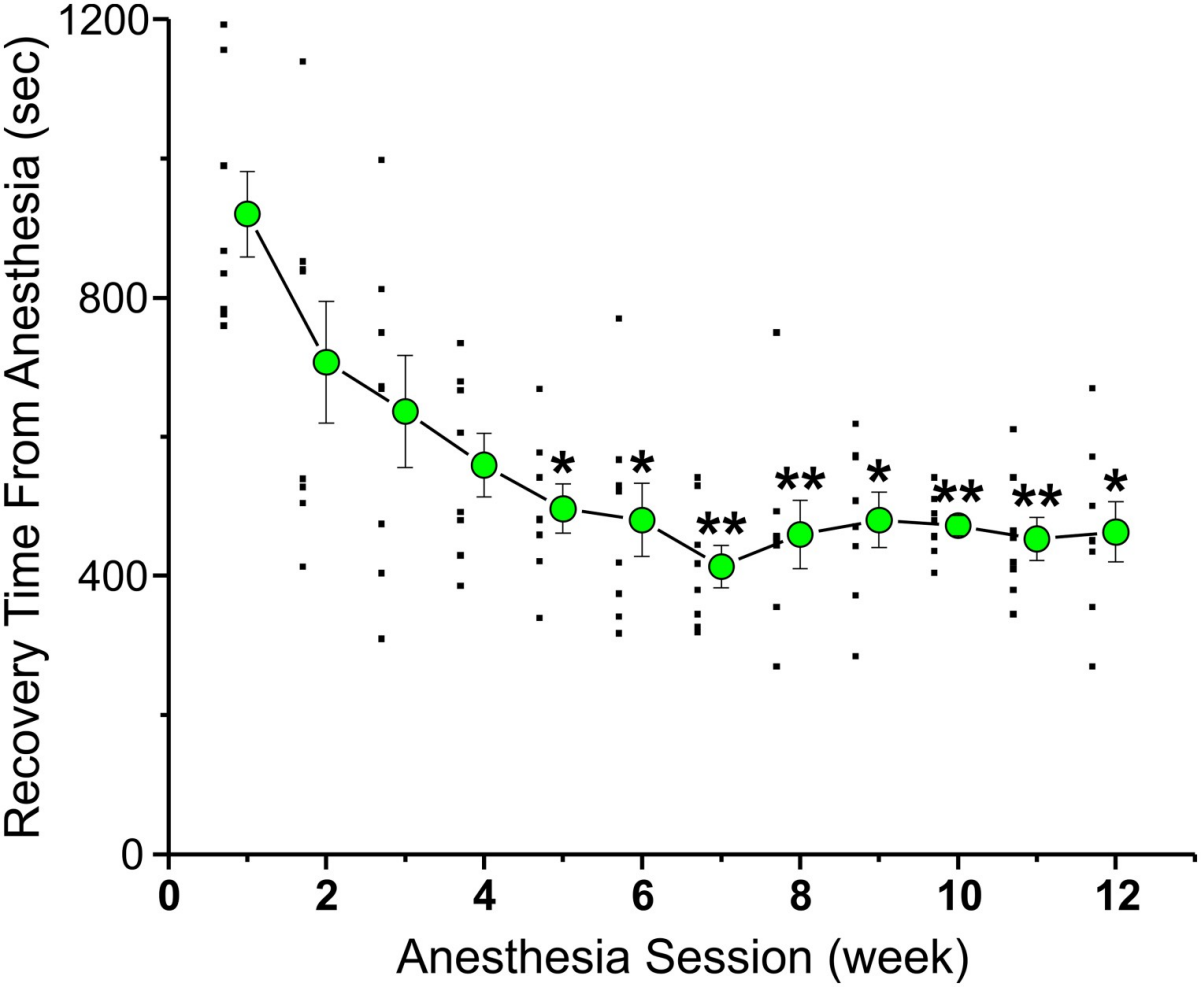
Decreased recovery times were observed after repeated handling and anesthesia sessions. A cohort of 8 adult rats was anesthetized once a week, for 12 weeks, with 2.5% isoflurane (4 L/min O2) for 1 hour in an anesthesia machine. The figure plots the average time it took for the rats to emerge from anesthesia (large filled green circles), every week. Each average from weeks 2–12 was compared to that obtained in week 1, using a repeated measure ANOVA. The “*” corresponds to a significant difference in the average values, p <0.05. The “**” denotes p<0.01. The raw data is plotted just to the left of each average as small filled black squares.

Emergence from anesthesia was tested in rats repeatedly exposed to anesthesia. Fig 2 plots data from a cohort of 8 male rats that were anesthetized weekly for 12 weeks (Group 1–12 total anesthesia sessions). The rats were exposed to 2.5% isoflurane with O_2_ 4 L/min for 1 hour inside a four chamber anesthetizing apparatus where 4 rats were anesthetized in 4 individual boxes at the same time. Recovery time from anesthesia for rats is defined as the time from when the animals are removed from the anesthesia chamber to when they were able to stand upright with 4 paws on the table. Fig 2 plots, as large green circles, the average recovery time from anesthesia of Group 1 rats, anesthetized 12 times. The raw data is plotted as small filled squares to the left of the average data points. Each average recovery time from weeks 2–12 was compared to that obtained in week 1, using a repeated measure ANOVA. After the 5^th^ anesthesia session the recovery time from anesthesia was already significantly faster than that observed on the first session. Accelerated recovery from anesthesia was observed for all subsequent anesthesia sessions. In this study, recovery time decreased by ~50%, a large and significant difference.

**Fig 3:**
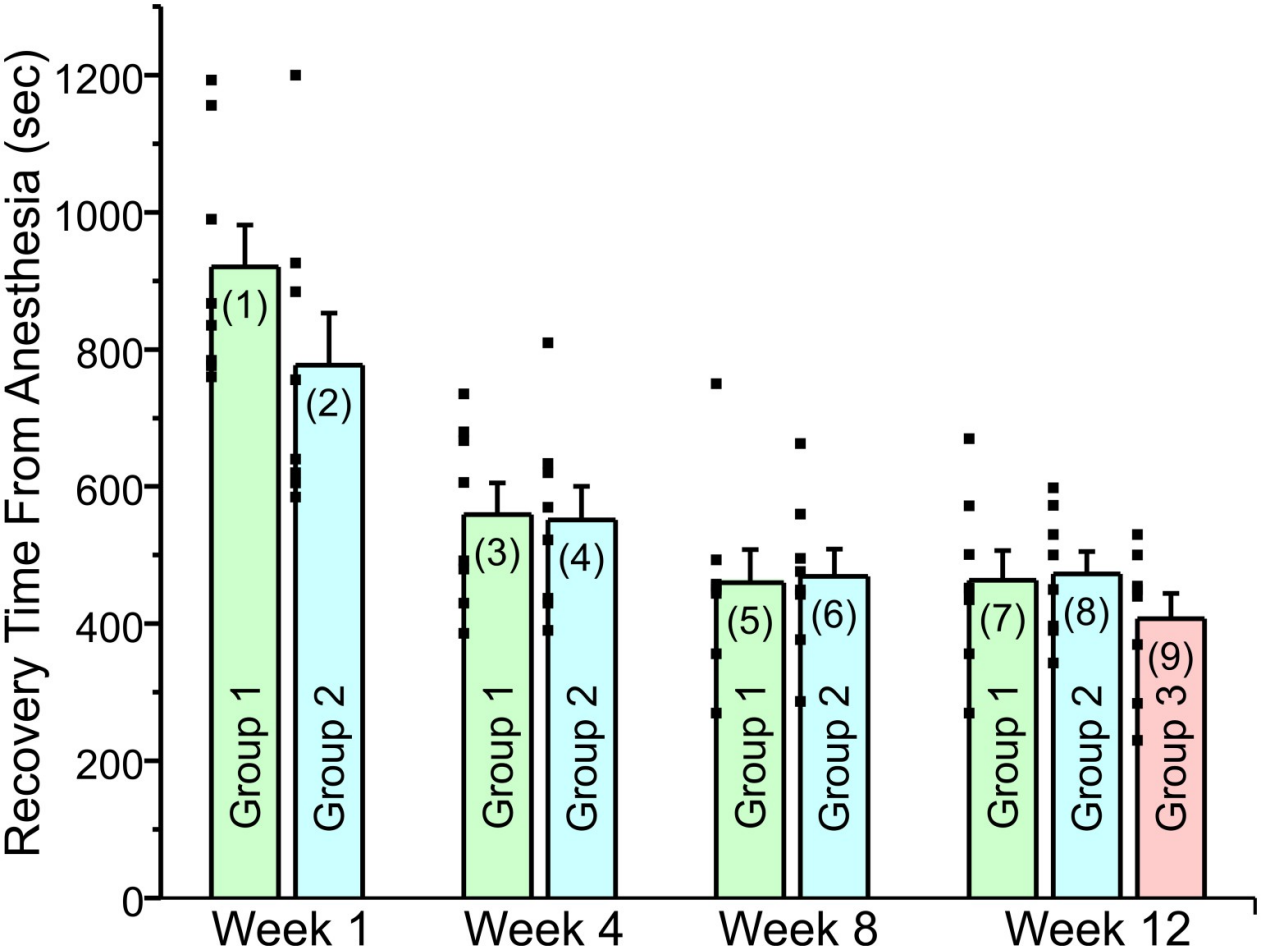
Weekly anesthesia or anesthesia on a 3 or 4 week schedule had similar effect on recovery time from isoflurane anesthesia as long as the rats were handled and transported on a weekly basis. **This was also true for rats that were anesthetized once, at week 12**. The figure plots the recovery time from anesthesia for the rats receiving weekly anesthesia, anesthesia on a 3–4 week schedule or receiving anesthesia once at week 12. The individual recovery times are plotted as small filled squares to the left side of each bar. All three groups were handled and transported every week. The figure shows the same time course for acceleration of recovery from anesthesia, without regard to whether the animals were anesthetized or not. Weekly anesthesia rats (Group 1) received isoflurane anesthesia once per week until week 12. Rats anesthetized on either a 3 or 4 week schedule, (Group 2) received isoflurane anesthesia at week 1, 4, 8 and 12. The rats in Group 3 were transported and handled in an identical manner to weekly rats and were anesthetized once at the very end of the study (week 12 Each data bar is labeled with a number in parenthesis. Recovery time, in seconds, for Group 1 on Week 1 is shown in Bar (1): Mean = 920 ± 61.2 (SEM). Bar (2): Mean = 777 ± 75.9. Bar (3): Mean = 559.5 ± 45.7. Bar (4): Mean = 551.6 ± 48.8. Bar (5): Mean = 459.5 ± 48.6. Bar (6): Mean = 468.8 ± 39.9. Bar (7): Mean = 463.3 ± 43.5. Bar (8): Mean = 472.6 ± 32.6. Bar (9): Mean = 407.4 ± 37. A paired T-test was used to compare data within groups while an independent T-test was used to compare data between groups. All comparisons can be found in Table 1. (1) vs (2) compares the significance of the difference between the data plotted in bar (1) vs bar (2). (1) vs (3) compares the significance of the difference between the data plotted in bar (1) and bar (3) etc. There are discrepancies in P value for Group 1 in Fig 2 and Fig 3 because repeated measure ANOVA and paired T-test were used respectively.

On its face, Fig 2 appears to suggest that repeated exposure to an anesthetic agent results in accelerated recovery from anesthesia which may indicate a diminished response to the isoflurane. Fig 3 shows that this is not the case. This figure includes rats that were transported from their homeroom to the anesthesia room on a weekly basis, but were only anesthetized on a 3 or 4 week basis (Group 2—total of 4 anesthesia sessions). Group 2 exhibited a similar acceleration in recovery time from anesthesia. Another group of rats (Group 3) was also transported on a weekly basis from their homeroom to the anesthesia room but were only anesthetized once, at the end of the 12th week study period. Rats in groups 2 and 3 were also placed in the anesthesia chamber with O_2_ for 5 minutes weekly when they were not scheduled to receive anesthesia. The purpose of this protocol was to mimic as closely as possible the handling of group 1 rats. Both groups 2 and 3 showed a comparable change in recovery time. These latter results suggest that the stress of handling and transportation resulted in the accelerated recovery from anesthesia.

**Table 1:** Comparing groups in Fig 3.

	(2)	(3)	(4)	(5)	(6)	(7)	(8)	(9)
(1)	= 0.164	= 0.003	<0.003	<0.002	<0.00003	<0.0006	<0.00002	<0.000005
(2)		= 0.028	<0.004	<0.004	<0.002	<0.003	<0.003	<0.0004
(3)			= 0.91	= 0.15	= 0.16	= 0.016	= 0.14	= 0.02
(4)				= 0.2	= 0.01	= 0.2	= 0.09	= .03
(5)					= 0.89	= 0.94	= 0.83	= 0.41
(6)						= 0.93	= 0.88	= 0.28
(7)							= 0.87	= 0.34
(8)								= 0.21

**Fig 4:**
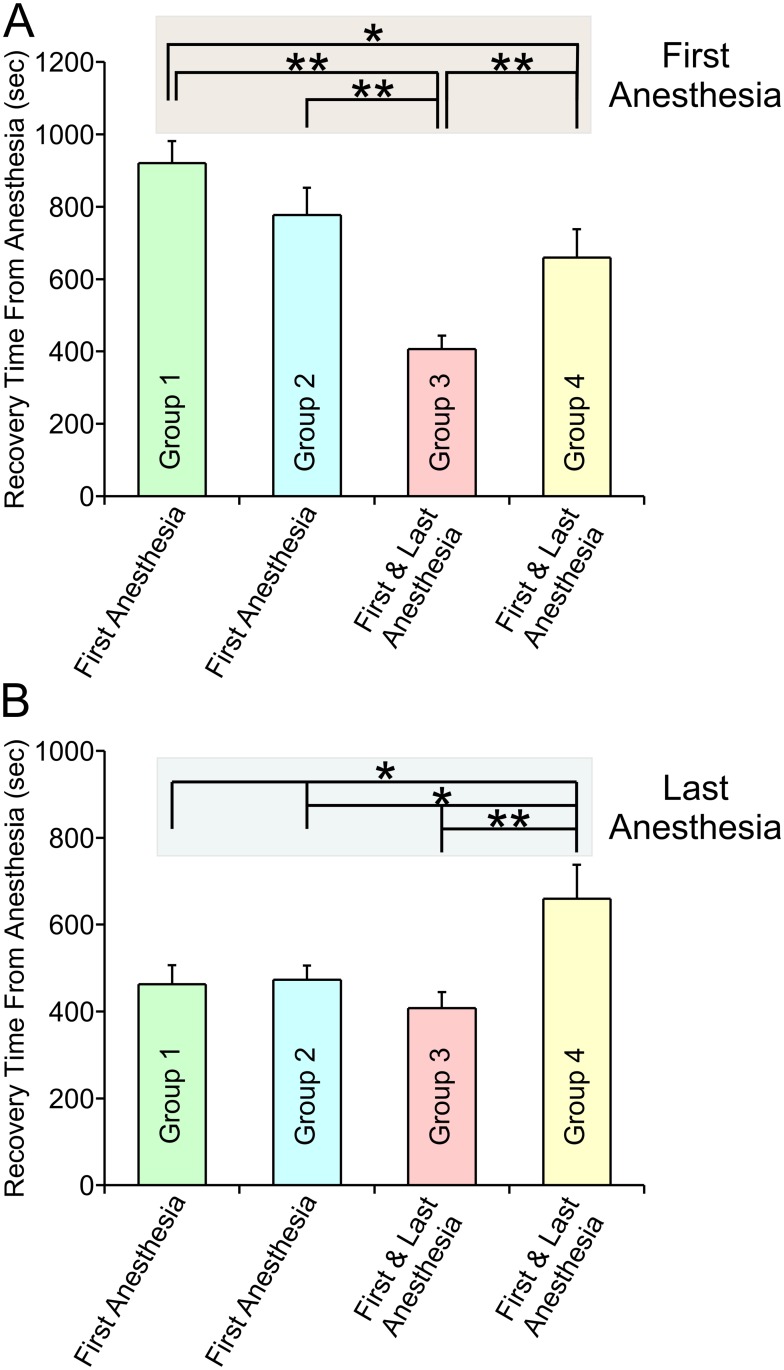
Comparison of the recovery times from both the first and last anesthesia sessions for Groups 1–4 shows that rats that were never transported or handled previously recovered slowly from anesthesia while rats that had been transported and handled recovered quickly. A, Plots the mean recovery time for all 4 groups on their first anesthesia sessions. Group 1 rats (green bars) were transported every week for 12 weeks and were anesthetized each time. Plotted is the mean recovery time from the week 1 anesthesia session (week 1: Mean = 920 ± 61.2 (SEM)). Group 2 rats (cyan bars) were transported every week for 12 weeks but were anesthetized 4 times, at weeks 1, 4, 8 and 12. Plotted is the mean recovery time from the week 1 anesthesia session (week 1: Mean = 777 ± 75.9). Group 3 rats (pink bar) were transported every week for 12 weeks but were anesthetized once, at week 12. Plotted is the mean recovery time from the week 12 anesthesia (week 12: Mean = 407.4 ± 37). Group 4 rats (yellow bar) were never transported until week 12, when they were transported and anesthetized once. Plotted is the mean recovery time from the week 12 anesthesia (week 12: Mean = 659.6 ± 78.2). B, Plots the mean recovery time for all 4 groups on their last anesthesia sessions. Group 1: Mean = 463.3 ± 43.5. Group 2: Mean = 472.6 ± 32.6. Group 3: Mean = 407.4 ± 37. Group 4: Mean = 659.6 ± 78.2. Note that for Groups 3 and Group 4 which received a single round of anesthesia, the first and last anesthesia session are the same. Raw data: The individual recovery times for Groups 1, 2 and 3 at week 1 and week 12 are plotted in Fig 3 as small filled black squares. For group 4 the recovery times (in sec) at week 12 are: 557, 974, 525, 687, 754, 449, 382 and 949. A paired T-test was used to compare data within groups while an independent T-test was used to compare data between groups. All comparisons can be found in the Tables 2 and 3.

If this hypothesis is true, then rats that were never stressed by transportation and handling, but which were kept in their home cages throughout the same 12 week period should exhibit a slow recovery from anesthesia. That was indeed the case. Fig 4 plots the average recovery time for all 4 groups on their first (Fig 4A) and last anesthesia sessions (Fig 4B). Examining the first anesthesia sessions exclusively, group 1, 2 and 4 rats were never transported or handled prior to their initial anesthesia session. Note that the first anesthesia recovery times for these 3 groups were significantly slower (Group 1–920 ± 61.23, n = 8; Group 2–777 ± 75.9, n = 8; Group 4–659.6 ± 78.2, n = 8) than the recovery time on the first anesthesia session for Group 3 rats (Group 3–407.4 ± 37, n = 8, p<0.000005, p<0.0007 and p<0.01, respectively) (Fig 4A). Group 3 rats were transported and handled 12 times prior to their initial anesthesia session. These results suggest that it is the stress associated with transportation and handling that results in the changes in the recovery time observed.

**Table 2:** Comparing groups in Fig 4A. First Anesthesia.

	Group 2	Group 3	Group 4
Group 1	= 0.164	<0.000005	= 0.04
Group 2		<0.0007	= 0.3
Group 3			<0.01

**Table 3:** Comparing groups in Fig 4B. Last Anesthesia.

	Group 2	Group 3	Group 4
Group 1	= 0.87	= 0.34	= 0.02
Group 2		= 0.21	= 0.01
Group 3			= 0.004

Examining the final anesthesia sessions for Groups 1–4, Fig 4B plots recovery times from anesthesia. Groups 1–3 were transported and handled 12 times on a weekly basis prior to the last anesthesia. These 3 groups had very similar recovery times. Group 4 was never transported or handled prior to its last anesthesia session. In this group, emergence in Group 4 was significantly slower than that of Groups 1–3. Recovery time for Group 4 was significantly different from that of Group 1, 2 or 3 (659.60 ± 78.20 vs 463.25 ± 43.50, n = 8, p = 0.02; 659.6 ± 77.20 vs 472.63 ± 32.65, n = 8, p = 0.01; 659.6 ± 78.20 vs 407.38 ± 37.04, n = 8, p = 0.004 respectively). Note that for groups 3 & 4, the last anesthesia session was also the first anesthesia session. This data is consistent with the hypothesis that stress alters the operation of the anesthetic agent.

**Fig 5:**
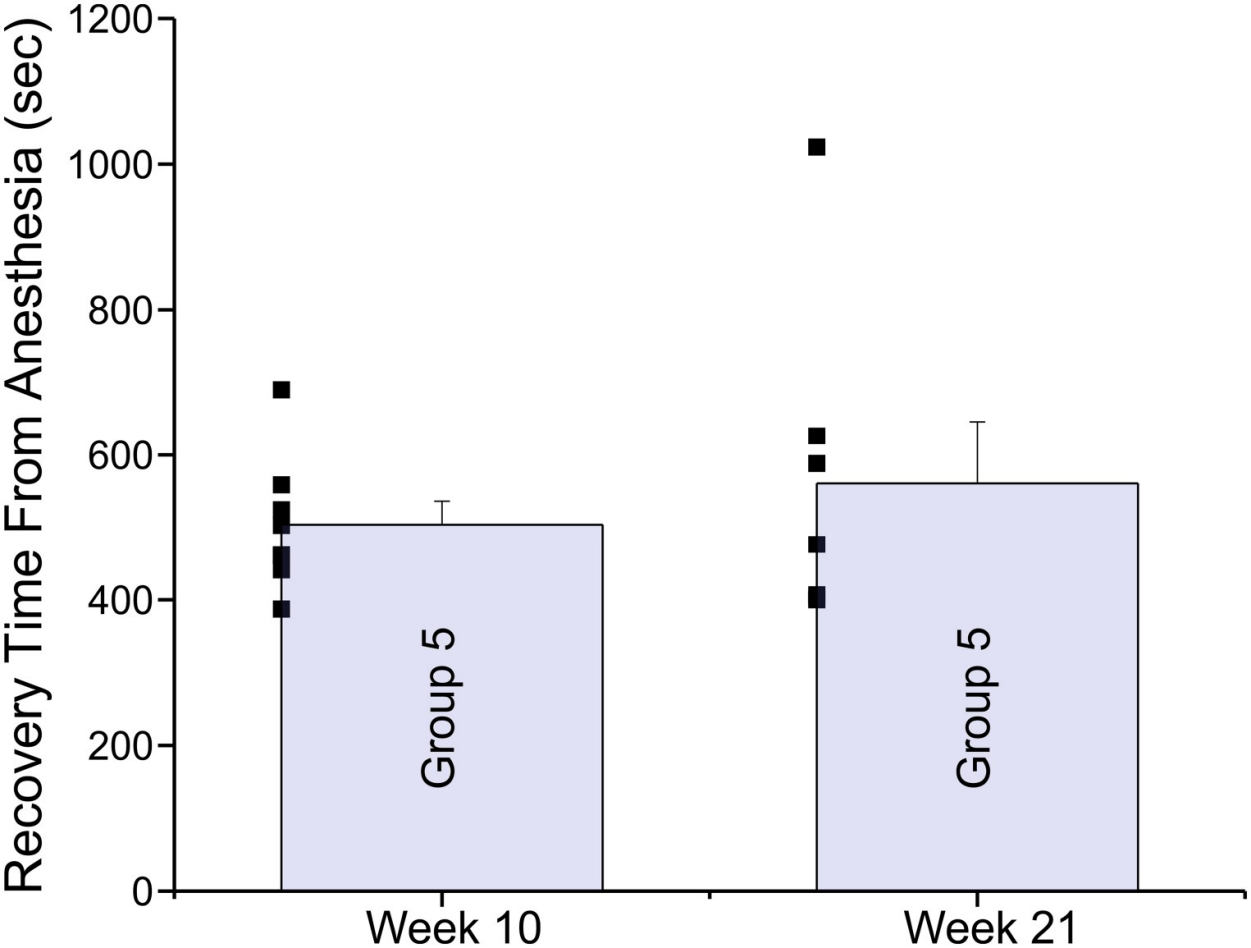
The acceleration in emergence from anesthesia was maintained even after an 11 week hiatus where rats were not transported or handled in any manner. This group of 8 rats was anesthetized with 3% isoflurane for 60 minutes (4 L/ min O_2_), ten times (weeks 1–10). At that time the animals were left untouched for the following 11 weeks (hiatus). Plotted are the average recovery times observed at week 10 (504 ± 32.4), prior to the hiatus and at week 21 after the 11 week hiatus (560.9 ± 84.6). Recovery times were not different after the hiatus when compared to before the hiatus (p = 0.52). The raw data is plotted as a scatter to the left of each bar chart.

Fig 5 plots data from a different cohort of 8 rats. In this experiment, rats were anesthetized every week for 10 weeks (3% isoflurane for 1 hour) and were then kept in their homerooms with no transportation or handling for a further 11 weeks, in order to determine whether any recovery would take place. Shown in Fig 5 is the average recovery time from anesthesia on week 10 (10^th^ anesthesia) and week 21 (11^th^ anesthesia). The 11-week hiatus in testing produced little recovery, although the scatter data suggests that a single animal may have recovered. These results suggest that the changes produced by stress are long lasting and were not readily reversed even after an 11-week hiatus period where the rats were not transported, handled or anesthetized in any manner.

In a final series of experiments, multiple exposures to 2% isoflurane for 1 hour also produced an acceleration in recovery time, similar to that described for 2.5% and 3% isoflurane (using a small cohort of 4 animals, 2% isoflurane was tested. In this experiment 2 weeks of data were combined to get 8 recovery times per group. So week 1 and 2 were combined. As was week 3 and 4 etc. For weeks 1 + 2 the average recovery time in seconds was 649.8 ± 38.8 (SEM) while for weeks 7 and 8 the average recovery time was 392.4 ± 50.3. The difference was significant (p = 0.01).

[Week 1 recovery times: 744, 447, 672, 713—Week 2 recovery times: 510, 733, 683, 696
Week 7 recovery times: 305, 430, 400, 282—Week 8 recovery times: 482, 272, 685, 283]

No adverse events were observed by us or reported by the staffs in the animal care facility.

## Discussion

The purpose of this study was to determine whether multiple exposures to an anesthetic agent, isoflurane and/or the stress involved in multiple exposures, could alter subsequent responses to that same agent. Recovery of consciousness from isoflurane anesthesia was dramatically faster in animals that were exposed to anesthetic multiple times than in control rats, possibly due to a change in sensitivity to the anesthetic agent. Surprisingly, the agent leading to the changes in subsequent responses to anesthetics was the transportation and handling the animals on a weekly basis. Rats that were anesthetized less frequently showed the same accelerated recovery from anesthesia, as long as they were transported and handled on a weekly basis. Indeed, even animals that were anesthetized just once showed the same recovery time, as long as they were transported and handled on a weekly basis (Fig 3). Figs 4 and 5 show that prior to transportation or handling, rats recovered slowly from anesthesia.

The conclusion put forward in this study, that cumulative stress produced by repetitive transportation and handling altered anesthetic efficacy. However, the opposite conclusion is also possible. It may be that the first transportation and handling was the most stressful and that the rats adapted to this stress over time. This would imply that the results from the first anesthesia sessions were aberrant and that later sessions were more representative of emergence from anesthesia times. In accord with this hypothesis, studies from other labs have shown that rats adapt to stressful procedures over time [16, 17]. Although possible, we don’t believe this to be the case because rats exposed to different forms of stress, like daily gavage of saline in a different study, exhibited the same accelerated recovery from anesthesia (first anesthesia after 60 days of oral gavage with 2% isoflurane for 90 minutes: mean recovery time 480 ± 26.2 (SEM), combining 2 weeks of data (n = 16)). It seems less likely that adapting to one form of stress, daily gavage, would also produce an adaptation in a different form of stress (transportation/ handling/ anesthesia). Nonetheless, we cannot rule out this possibility.

[Oral gavage week 1 recovery times: 541, 697, 395, 523, 524, 348, 363, 390
Oral gavage week 2 recovery times: 395, 600, 438, 590, 421, 511, 363, 580]

In addition to the weekly handling and transportation, sleep patterns in Groups 1–3 rats were disrupted. Rat are nocturnal animals; they tend to sleep during the day and stay awake at night [18, 19]. All our experiments were performed in the daytime. Sleep disturbance may add to the stress levels in these rats.

In a study examining long-term exposure to anesthetics, Smith et al. (1979) [20] suggested that tolerance and dependence of inhalational agents could occur after chronic exposure to these anesthetics. However, it is difficult to compare that study to this study since they used very different protocols. Animals were exposed to inhalational agents for much longer durations than were used in this study and the concentrations of inhalational agents in their study were much lower (subanesthetic concentrations). These differences make direct comparisons problematic. There is one aspect of Fig 4A that appears inconsistent. Prior to their first anesthesia, Groups 1, 2 and 4 were never transported or handled. Fig 4A shows that emergence from anesthesia was somewhat more rapid in Group 4 than it was in Group 1 (Group 1–920 ± 61.23 sec: Group 4–659.6 ± 78.20 sec, p = 0.04). This difference is most likely due to variability between groups, something we have previously observed [15]. In fact, recovery time for Group 2 was intermediate between that of Groups 1 and 4 (Group 2–777 ± 75.9). Other possibilities are that the difference is due to either the age or the weight of the rats. Groups 1–2 rats were ~3 months old and weighed ~ 350 grams when they were transported, handled and anesthetized the first time. In contrast, Group 4 rats were ~6 months old and weighed ~550 grams when they were transported handled and anesthetized the first time. Fig 2 suggests that the weight of the rats is unlikely to play an important role in recovery times, since most of the change in recovery times occurred within a few weeks, whereas most of the change in weight occurred during the period of time when recovery times were relatively stable (weeks 4–12). In addition, the average weights among all 4 groups in week 12 were not different. Chemali et al. [21] showed a dramatic increase in recovery time when comparing young adult and aged rats. Even so, it is difficult to compare our data to that of Chemali et al., as their study compared rats 6–8 months old with rats more than 2 years old whereas our study compared rats ~3 months old with those ~6 months old. It is likely that most of the effect observed in the Chemali et al. [21] study takes place in rats that are already quite old.

Our results suggest that stress-related acceleration in recovery from isoflurane anesthesia was persistent. Even after an 11week hiatus from anesthesia exposure, emergence from anesthesia was dramatically accelerated (see Fig 5).

Multiple exposures to 2% or 3% isoflurane for 1 hour produced a similar acceleration in recovery from anesthesia. The change in recovery from anesthesia occurred in all three concentrations of isoflurane used in this study. However, the percentages of change among three different concentrations were difficult to compare because the dose response curve could not be performed in the same group of animals.

Volatile anesthetics have many clinical effects, including two main ones: unconsciousness and immobility [4]. Most studies employing anesthesia examine the minimum alveolar concentration (MAC) for immobility. In these studies, 1 MAC is defined as the end tidal concentration of inhalational anesthetic in the lungs needed to prevent a motor response in 50% of subjects in response to a painful stimulus, like surgery [22]. Age-related changes in MAC have been better studied in humans [22] and in rats [23, 24]. MAC for most inhalational agents seems to be relative stable in adult humans and rats, but declines significantly in the later stage of their life. MAC for recovery of consciousness from general anesthesia in rats with different ages is not as well studied as MAC for immobility. The mechanism for anesthetic action on immobility may be different from that for unconsciousness. Recovery time from anesthesia was the time from when the animals were removed from the anesthesia chamber to when they stood with 4 paws on the table. Although the rats were still staggering, at recovery the animals had regained significant motor control. This is closest to a different measure of anesthesia called MACawake [25], which is defined as the end-tidal concentration of inhalational anesthetic that prevents appropriate voluntary responses to spoken commands, like “open your eyes”, in 50% of subjects [26]. Anesthetic levels at MACawake are lower than they are for a standard MAC. Evidence in the literature suggested the effect of anesthetic on immobility is mainly due to its action in the spinal cord while its action on consciousness is mainly in the brain [27].

A large number of studies have explored, stress-induced changes, which has led to the conclusion that stress deleteriously affects health and alters neuronal function. Although there is still controversy surrounding the meaning of stress, available data strongly suggest that stress is linked to the etiology and progression of disease. Chronic stress can result in anxiety, insomnia, high blood pressure and a weakened immune system [5]. The brain is the key organ in the response to stress [10]. The developing brain is particularly vulnerable to stress and stress can alter the architecture of the developing brain [6]. At all ages, stress alters brain architecture, gene expression and function [28]. Elevated levels of corticosterone, the major stress hormone, produces persistent cognitive and emotional changes [10]. Memory appears to be especially prone to disruption by stress [11]. The hippocampus shows a loss of connectivity and synaptic function in response to stress, which may explain the alterations in memory observed [10]. There may be a loss of neurons in the human hippocampus in response to stress [29]. Even short-term stress, lasting just a few hours, impairs synaptic communication and may lead to altered learning and memory [7]. Repeated chronic stress leads to cognitive impairment by altering glutamate receptor expression [8] and memory alterations may be produced by altered long-term potentiation [9]. Stress responses may trigger mental illness [12]. Chronic stress may lead to fewer neurons being produced and elevated numbers of glial cells; this imbalance may lead to mental disorders [13].

Although there is a more limited literature regarding the effects of stress on drug responses, there are strong indications that these too are altered by stress in rats. In the offspring of gestating mothers exposed to daily foot shocks, the offspring showed altered responses to both nicotine and benzodiazepine withdrawal [30] and the animals were predisposed to nicotine addiction [31, 32]. Furthermore, the animals exhibited differences in 5-HT_1A_ and D_2_ dopamine receptor expression, which suggest that drugs that interact with these receptors may have altered responsiveness [31, 32]. The effects of prenatal stress can be long lasting. For instance, the alterations in benzodiazepine sensitivity produced by prenatal stress lasts into adulthood [33]. In animals of all ages, stress induced by physical restraint altered responses to ethanol, possibly via alterations in GABA_A_ receptor systems [34]. This brief review suggests that stress can alter responsiveness to drugs and that the effects are persistent. The literature outlined above is consistent with our own findings that stress changed the responsiveness to isoflurane and that the change was maintained. Furthermore, isoflurane shares biological targets with two of the drugs outlined above and which may be altered by stress. For instance, isoflurane potentiates GABA_A_ receptor activity [35] as does ethanol [36] and benzodiazepines [37]. It is tempting to speculate that the observed alteration of the response to isoflurane may be due, at least in part, to changes in GABA_A_ receptor expression or function or alternatively some alteration in the production of the neurotransmitter itself. If true, then intravenous anesthetics like etomidate and propofol will be even more susceptible to alteration by stress, as GABA_A_ receptors play an even larger role in their mechanism of action [38] than they do for inhalational agents like isoflurane.

Not surprisingly, surgery and anesthesia appear able to activate the body’s response to stress [14]. To reduce the perioperative stress for patients, it is a common practice clinically that patients receive anxiolytic in the preoperative period. It is known that repeated exposures to narcotic or benzodiazepine may lead to tolerance of subsequent use of these drugs in the same patient [1–3, 39]. The mechanisms of volatile anesthetics are less understood [27, 35]. Evidence in the literature suggest multiple exposures to anesthetics may lead to neuroapoptosis and cognitive dysfunction in developing brain [40–42]. The FDA recently issued a warning about the multiple uses of anesthetics, including volatile anesthetics and their potential long term side effects in young children (https://www.fda.gov/downloads/Drugs/DrugSafety/UCM554644.pdf). The impact of stress related to anesthesia and surgeries were not as well studied as the direct effects of anesthetics in the brain in animal and human studies. Recent study showed multiple volatile anesthetic exposures in infant monkeys increased the frequency of anxiety-related behaviors later in life of these animals [43]. The direct cause of the increased anxiety was not known.

In this study, we created an environment for the animals which was similar to patients who would undergo multiple anesthesia and stressful conditions like the preparation for multiple procedures. We show that repeated stress caused by transportation and handling, not repeated use of isoflurane itself, is the dominant factor in altering recovery from anesthesia. Further studies are required to measure levels of stress by using stress markers and determining whether those markers correlate with the degree of acceleration in recovery from anesthesia. It would also be interesting to examine whether people or animals that are exposed to a high stress environment, may become more resistant to anesthesia.
